# Movement matters: short-term impacts of physical activity on mood and well-being

**DOI:** 10.1007/s10865-023-00407-9

**Published:** 2023-03-20

**Authors:** Loree T. Pham, Raymond Hernandez, Donna Spruijt-Metz, Jeffrey S. Gonzalez, Elizabeth Ann Pyatak

**Affiliations:** 1 Mrs. T.H. Chan Division of Occupational Science and Occupational Therapy, University of Southern California, 1640 Marengo St., Suite 411, Los Angeles, CA 90033 USA; 2 Dornslife Center for Economic and Social Research, University of Southern California, Los Angeles, USA; 3 Keck School of Medicine, University of Southern California, Los Angeles, CA USA; 4 Department of Psychology, University of Southern California, Los Angeles, CA USA; 5 Ferkauf Graduate School of Psychology, Yeshiva University, Bronx, CA USA; 6 Division of Endocrinology, Department of Medicine, Albert Einstein College of Medicine, Bronx, NY USA

**Keywords:** Ecological momentary assessments, Accelerometry, Physical activity, Mood, Type 1 diabetes

## Abstract

Few studies have investigated the short-term, momentary relationships between physical activity (PA) and well-being. This study focuses on investigating the dynamic relationships between PA and affective well-being among adults with type 1 diabetes. Participants (*n* = 122) wore an accelerometer and completed daily EMA surveys of current activities and affective states (e.g., happy, stressed, excited, anxious) via smartphone over 14 days. Within-person, increased sedentary time was associated with less positive affect (*r* =  − 0.11, *p* < 0.001), while more PA of any intensity was associated with greater positive affect and reduced fatigue, three hours later. Between-person, increased light PA was associated with increased stress (*r* = 0.21, *p* = 0.02) and diabetes distress (*r* = 0.30, *p* = 0.001). This study provides evidence that positive affect and fatigue are predicted by previous activity regardless of the different activities that people engaged in. Positive affect increased after engaging in PA. However, participants with higher amounts of light PA reported higher stress ratings.

## Introduction

Physical activity is associated with numerous and substantial health benefits (Warburton & Bredin, 2017; Warburton et al., 2006), but while the prevalence of meeting high aerobic physical activity guidelines has increased over the past decade (Whitfield et al., 2021), only 53.3% of U.S. adults meet aerobic physical activity guidelines (CDC/National Center for Health Statistics, 2021). Excessive sedentary behavior may influence the development of chronic conditions such as cardiovascular disease (Chomistek et al., 2013; Patterson et al., 2018), type 2 diabetes (Bowden Davies et al., 2018; Paing et al., 2018; Patterson et al., 2018; Sardinha et al., 2017), certain cancers (Cong et al., 2014; Schmid & Leitzmann, 2014), and low back pain (Citko et al., 2018; Subramanian & Arun, 2017). It is especially important for individuals with type 1 diabetes to engage in physical activity (Riddell et al., 2017) as a means of reducing the risk of complications and comorbid conditions. Hence, there continues to be a need for the promotion of physical activity and an effort to reduce physical inactivity and sedentary activity.

Besides reducing the risk of chronic health conditions, there is substantial, evidence-based support that physical activity improves psychological well-being (Warburton & Bredin, 2017). While numerous behavior change theories (e.g. Michie et al., 2014) help to explain why people *adopt* physical activity behaviors, it is equally if not more important to consider what may help people *sustain* behaviors over time. For example, people are more likely to maintain behavior change if they are satisfied with the new behavior after weighing the relative costs and benefits (Rothman et al., 2011). Therefore, it is important to consider physical activity’s impact on mood and well-being, as the promotion of short-term positive mood states (Shiota et al., 2021; Van Cappellen et al., 2018) and well-being variables (Razazian et al., 2020; Stults-Kolehmainen & Sinha, 2014) may be beneficial in promoting sustainable long-term behavior change. Similarly, considering activity type and its importance to the individual may be useful when aiming to get people to move more, since certain activities may hold more meaning and impact compared to others. For instance, men spend a greater amount of time engaged in physical activity than women (Hamrik et al., 2014; Saffer et al., 2013), particularly in group-based occupations (e.g., sports), while women are more likely to participate in individual-level occupations (e.g., walking or housework) (Azevedo et al., 2007). Contextual factors may also play a role (Craft et al., 2014). For example, women and older adults are more likely to exercise at home, men are more likely to exercise outdoors or at work, younger adults are more likely to exercise with friends, and individuals with lower income are more likely to exercise with family members (Dunton et al., 2008; Welk & Kim, 2015). Gaining a better understanding of the impacts on mood may be beneficial in promoting participation in physical activity and improving health and well-being.

Though there exists an extensive body of research related to physical activity, there are a number of methodological limitations that need to be addressed (Kanning et al., 2013). For example, a vast majority of physical activity studies rely on retrospective self-report, such as estimating the amount of physical activity engaged in over the past week or month, which may be subject to participant bias and overestimation and may not be reliable (Warburton & Bredin, 2017). Some of these limitations may be addressed through the use of electronic ecological momentary assessment (EMA). EMA allows participants’ behaviors and experiences to be recorded in real-time and in real-world contexts while minimizing retrospective recall and memory biases (Shiffman et al., 2008). Using electronic EMA may be even more advantageous than the traditional paper-and-pencil EMA in terms of reliability and compliance (Berkman et al., 2014; Stone et al., 2002). An additional benefit of using electronic EMA is the ability to record the exact time of survey completion, which is beneficial when pairing the EMA data with other types of data, such as accelerometry data, that is collected in real time.

Overall, while there are numerous studies examining the relationship between physical activity and well-being (Marquez et al., 2020), relatively few studies have investigated the short-term, momentary relationships between the two. Furthermore, to our knowledge, activity type and activity importance have not been thoroughly explored in physical activity research. Lastly, there is a need to incorporate underrepresented populations, such as diverse racial and ethnic groups, in order to have a greater public health impact (Marquez et al., 2020). The study sample consists of individuals with type 1 diabetes, who face the same barriers for physical activity as the overall population but who also may face different challenges and levels of healthcare support (Pyatak et al., 2014). To address the present research gaps, this study selected and recruited from clinical sites with a diverse patient population, and used real-time data collection via EMA and accelerometry to examine the relationships between physical activity and well-being. Combined, these momentary data collection methods may offer greater insight into the relationship between physical activity and well-being. The purpose of this study is to investigate the relationship between physical activity and subsequent mood, while controlling for the effect of activity type on mood.

## Methods

### Participants

This study is a secondary analysis from an overarching study investigating relationships between blood glucose, function, and emotional well-being in adults with type 1 diabetes (T1D) (Pyatak et al., 2021). In this case, function refers to self-reported daily life activity performance, objective cognitive function, and physical activity derived from accelerometers. Emotional well-being in this study refers to positive and negative affect, stress, and diabetes distress. Participants were recruited from clinical sites in Los Angeles and New York City metropolitan areas. Eligibility criteria were specified to ensure that participants were able to fully complete the study procedures and do not have any health conditions besides diabetes that could significantly impact blood glucose levels. Participants were required to be able to answer EMA surveys throughout the day, including while at work. Greater detail regarding the parent study methodology and research questions are outlined in (Pyatak et al., 2021). In the parent study, 196 participants were recruited and started the study protocol.

## Study protocols

### Data collection

Due to COVID-19, recruitment and data collection was completed remotely, through phone calls, videoconferencing, emails, and mailings. Participants who expressed interest in the study completed a screening questionnaire over the phone. Eligible participants completed enrollment paperwork and baseline surveys through an online data capture platform, Research Electronic Data Capture (Harris et al., 2009). Study equipment was shipped directly to participants and included the following: accelerometer, smartphone, participant manual, ClinCard for loading stipends onto, and shipping materials to mail the study equipment back after the completion of data collection.

### Accelerometry

The Actigraph, Inc., wGT3X_BT model accelerometer was used to objectively measure participants’ physical activity, providing continuous data that could be used to infer time spent in sedentary, light, moderate, and vigorous physical activity each day. This core study device was attached to an adjustable strap, which participants wore on their non-dominant wrist. Participants were instructed to wear the device continuously for 14 consecutive days, removing the device only for water-based activities, such as bathing. The accelerometer recorded the intensity of participants’ activity at a 30 Hz sampling frequency, with each recording time-stamped. The 30-s epoch was chosen over longer epochs in order to better capture shorter bouts of activity, which may help to reduce misclassification errors of physical activity estimates (Brønd & Arvidsson, 2016; Gabriel et al., 2010).

### Ecological momentary assessment (EMA)

Participants completed 14 days of EMA surveys and mobile cognitive tasks via Xiaomi Mi A1 smartphone (AT&T USA, Inc.). The phones came pre-installed with the necessary apps, including mEMA (mobile EMA by Ilumivu) which is a HIPAA-compliant software application that would prompt the EMA surveys at 3-h intervals and store survey responses on a secure cloud-based server for data management (ilumivu: Software for Humanity, n.d.). Participants completed about 5–6 surveys per day that were scheduled depending on the participants’ individual preferences. Depending on the branching logic, participants answered approximately 30 survey items in the first five surveys of the day and 50 items in the evening survey.

### Measures

#### Physical activity

Using the data recordings from the accelerometer, activity counts were converted to minutes spent in light, moderate, and vigorous physical activity. Light physical activity was defined as the time that was not spent in either moderate-vigorous activity or sedentary activity (which is considered less than 100 counts/minute; Healy et al., 2008). Consistent with national surveillance studies (Troiano et al., 2008), moderate-vigorous physical activity is defined as 2020 activity counts per minute. Time in physical activity ranges were based on three hour periods with at least 75% of the data present in that period.

#### Well-being and activity

Positive affect, negative affect, stress, diabetes distress, and fatigue were assessed using EMA survey questions (Table 1). The EMA questions were selected based on validated global measures and/or being successfully used in previous EMA studies (Broderick et al., 2009; Crawford & Henry, 2004; Dunton et al., 2008; Laurenceau, 2013; Merwin et al., 2015; Scott et al., 2017). At each time point, participants rated indicators of well-being as well as identified the type of activity that they were engaging in at the time of the signal. In order to reduce selection and recall bias for activity type, a sampling approach rather than coverage approach was used, where participants selected the type of activity that they were immediately engaged in prior to the survey, rather than selecting the activity that they were engaged in for the longest duration in the time frame period to the survey notification (Shiffman et al., 2008). Possible activity type responses were derived from the *Occupational Therapy Practice Framework: Domain and Process-Fourth Edition* (American Occupational Therapy Association, 2020b) using lay language agreed upon by the research team with input from pilot testing of the study protocol prior to enrolling participants. The term “activity” was chosen as a more accessible term for the study participants, but the terms “activity” and “occupation” will be used interchangeably in the subsequent discussion.

**Table 1:** Ecological momentary assessment survey measures

Construct	Item(s)	Response Option(s)
*Well-Being*		
Positive affect	4 items: Average of mood ratings for “happy”, “content”, “enthusiastic”, “excited”	For each mood, 0 (not at all) to 100 (extremely)
Negative affect	4 items: Average of mood ratings for “tense”, “upset”, “sad”, “disappointed”	For each mood, 0 (not at all) to 100 (extremely)
Stress	How stressed are you right now?	0 (Not at all stressed) to 100 (Extremely stressed)
Diabetes distress	How stressed do you feel about your diabetes or diabetes management right now?	0 (Not at all stressed) to 100 (Extremely stressed)
Fatigue	At this moment, how tired do you feel?	0 (Not at all) to 100 (Extremely)
Pain	At this moment, how much bodily pain do you have?	0 (None) to 100 (Extreme pain)
Activity		
Activity type	What were you doing right before starting this survey?	Work/school activities (e.g., paid labor, volunteer work, and studying)*
*activity type examples were not included in the survey but were listed in the participant manual and elaborated upon during the baseline training		Traveling (e.g., driving, riding in a car, walking)
		Relaxing/chilling (e.g., passive leisure like watching Netflix, listening to music)
		Sleeping/napping
		Socializing (e.g., talking with friends/family)
		Caring for myself (e.g., eating, dressing, bathing, toileting, personal grooming)
		Caring for others (e.g., caring for your children and pets, if you’re caring for others as part of work this counts as “work”)
		Doing housework/errands (e.g., paying bills, washing dishes and clothes, exercising for health)
		Fun/play/leisure activities (e.g., active leisure like exercising for fun, video games, reading for fun)
		Other (If chosen, please specify)
Activity importance	How important is this activity to you?	0 (Not at all) to 100 (Extremely)

### Statistical analysis

In order to be included in the analysis, participants need to have worn the accelerometer for at least 75% of the time block (i.e. 2 h and 15 min out of 3 h). Between and within-person correlations between physical activity, well-being, and activity measures were calculated using the “psych” package in R (Revelle, 2021). The “statsBy” function in this package can decompose observed correlations from longitudinal data into within and between-person components, with the within portion derived from the pooled correlation within groups and between portion from the weighted correlation of the means between groups (Revelle, 2021). The correlations are between measures of well-being at the time of the survey and physical activity metrics calculated from the three-hour time period preceding the survey prompt.

To determine if relationships between physical activity and well-being were significant after adjusting for activity type and activity importance, mixed effects models were run using the “lme4” package in R (Bates et al., 2015). Compared to ordinary regression, mixed models are better suited for analyzing longitudinal data as they can account for non-independence of observations (e.g., multiple days nested in an individual) (Bell, 2013). Separate models were run, with positive affect, negative affect, stress, fatigue, or pain as the dependent variable, a single PA metric as the focal predictor (i.e. time sedentary, time light, or time moderate-vigorous), and activity type and/or activity importance as a covariate to control for it. Person-mean centered versions of the PA variables and activity type and importance were used to make sure that only their within-person components (and not between-person) would be represented (Curran & Bauer, 2011). In terms of the mixed model settings, the intercept and slopes of the PA variables were specified as random effects, which allows them to vary according to the clusters (e.g. different individuals). An unstructured covariance matrix was used, which chooses parameters that best fit the data at the cost of a greater number of parameters specified (Kincaid, 2005).

## Results

### Descriptive statistics

Of 136 total participants at the time analyses were conducted, 14 were excluded due to incomplete data, leaving A diverse sample of 122 total participants included in the analysis (see Table 2 for demographic characteristics). The median EMA completion percentage was 92.86%, and four or more EMA surveys were completed on 82.34% of all data collection days across the participants. The final dataset included a total of 8,639 EMA data points across 1,812 days.

**Table 2:** Demographic characteristics (*n* = 122)

Characteristic	*n*	Mean (SD) or Percent (%)
Age (years)	122	41.12 (14.83)
*Gender*		
Male	54	44%
Female	68	56%
*Race/Ethnicity*		
White	43	35%
Latino/x	46	38%
Black	14	11%
Asian	4	3%
Multi-ethnic	9	7%
Other	4	3%
Not provided	2	2%
*Education*		
High school grad or less	25	20%
Some college, no degree	26	21%
Associate’s degree	13	11%
Bachelor’s degree	37	30%
Graduate degree	19	16%
Not provided	2	2%
*Annual household income*		
Less than $50,000	47	39%
$50,000–$99,999	23	19%
Above $100,000	21	17%
Did not wish to provide	24	20%
Did not know	7	6%

### Relationships between physical activity and subsequent well-being

#### Within-person

Increased time spent sedentary over a three hour period was associated with decreased subsequent positive affect (*r* =  − 0.03, *p* < 0.01) and increased diabetes stress (*r* = 0.04, *p* < 0.001) three hours later. Meanwhile, more physical activity at any intensity was associated with greater positive affect and reduced fatigue three hours later. Similar increases in positive affect were observed with light, moderate, and vigorous PA (*r* = 0.10, 0.10, 0.09 respectively, all *p* < 0.001). Fatigue decreases with light PA, moderate PA, and vigorous PA (*r* =  − 0.07, − 0.05, − 0.05 respectively, all *p* < 0.001). See Table 3 for full results looking at positive and negative affect, stress, diabetes stress, fatigue, and pain at different activity levels. Based on the significant correlations seen with positive affect and fatigue, additional testing was run to determine if the correlations were due to any other factors, such as the activity just reported. Table 4 illustrates the relationship between physical activity and positive affect before and after adjustment by activity type and activity importance. Table 5 illustrates the relationship between physical activity and fatigue before and after adjustment by activity type and activity importance. Table 6 indicates the various activity types that were significantly associated with positive affect, when using the “relaxing/chilling” activity type as a reference point.

**Table 3:** Within-person relationships between physical activity and subsequent well-being

	Positive Affect	Negative Affect	Stress	Diabetes Stress	Fatigue	Pain
Sedentary	*r* = − 0.11**	*r* = 0.02	*r* = − 0.01	*r* = 0.02	*r* = 0.06***	*r* = − 0.01
Light	*r* = 0.10***	*r* = − 0.01	*r* = 0.01	*r* = − 0.02	*r* = − 0.07***	*r* = 0.01
Moderate	*r* = 0.10***	*r* = − 0.01	*r* = 0.02	*r* = − 0.02	*r* = − 0.05***	*r* = 0.02
Vigorous	*r* = 0.09***	*r* = − 0.02	*r* = 0.00	*r* = − 0.01	*r* = − 0.05***	*r* = 0.00
Moderate or Vigorous	*r* = 0.11***	*r* = − 0.02	*r* = 0.01	*r* = − 0.01	*r* = − 0.06***	*r* = 0.01

^*^ = significant at *α* = 0.05; ** = significant at *α* = 0.01; *** = significant at *α* = 0.001

**Table 4:** Within-person physical activity and positive affect after adjustment for activity type and activity importance

	Before adjustment		After adjustment	
For every one minute increase in time spent in **sedentary activity**, positive affect **decreased** by:	− 0.058	*p* < .001	− 0.038	*p* < .001
For every one minute increase in time spent in **light activity**, positive affect **increased** by:	0.193	*p* < .001	0.120	*p* < .001
For every one minute increase in time spent in **moderate or vigorous activity**, positive affect **increased** by:	0.069	*p* < .001	0.045	*p* < .001

**Table 5:** Within-person physical activity and fatigue after adjustment for activity type and activity importance

	Before adjustment		After adjustment	
For every one minute increase in time spent in **sedentary activity**, fatigue **decreased** by:	0.054	*p* < .001	− 0.007	*p* = 0.643
For every one minute increase in time spent in **light activity**, fatigue **increased** by:	− 0.230	*p* < .001	− 0.045	*p* = 0.395
For every one minute increase in time spent in **moderate or vigorous activity**, fatigue **increased** by:	− 0.065	*p* < .001	0.009	*p* = 0.629

**Table 6:** Within-person relationship between activity type and positive affect

		Estimate	*p*-value
Sedentary	Time spent	0.038	< 0.001
	Activity importance	0.17	< 0.001
	Activity reported (Relaxing/chilling is reference group)		
	Doing Fun/play/leisure activities	4.29	< 0.001
	Doing Sleeping/napping	− 7.04	< 0.001
	Doing Socializing	5.43	< 0.001
	Traveling	1.21	0.166
	Doing Work/school activities	− 2.41	< 0.001
Light	Time spent	0.120	< 0.001
	Activity importance	0.17	< 0.001
	Activity reported (Relaxing/chilling is reference group)		
	Doing Fun/play/leisure activities	4.33	< 0.001
	Doing Sleeping/napping	− 7.00	< 0.001
	Doing Socializing	5.34	< 0.001
	Traveling	1.34	0.126
	Doing Work/school activities	− 2.40	< 0.001
Moderate or vigorous	Time spent	0.045	< 0.001
	Activity importance	0.17	< 0.001
	Activity reported (Relaxing/chilling is reference group)		
	Doing Fun/play/leisure activities	4.35	< 0.001
	Doing Sleeping/napping	− 7.03	< 0.001
	Doing Socializing	5.55	< 0.001
	Traveling	1.28	0.143
	Doing Work/school activities	− 2.42	< 0.001

#### Between-person

When examining between-person relationships between physical activity and well-being, increased light PA was associated with increased stress (*r* = 0.21, *p* = 0.023). See Table 7 for full results. As we hypothesized this finding could be due to work demands that may be stressful and require light PA, we ran an additional test to determine if the increase in stress was moderated by employment status (i.e., working or not); but significant moderation was not found (*p* = 0.10).

**Table 7:** Between-person relationships between physical activity and subsequent well-being

	Positive affect	Negative affect	Stress	Diabetes distress	Fatigue	Pain
Sedentary	*r* = 0.14	*r* = − 0.09	*r* = − 0.10	*r* = − 0.10	*r* = 0.10	*r* = 0.05
Light	*r* = − 0.02	*r* = 0.13	*r* = 0.21*	*r* = 0.30**	*r* = 0.09	*r* = 0.13
Moderate	*r* = − 0.13	*r* = 0.10	*r* = 0.11	*r* = 0.20*	*r* = − 0.02	*r* = 0.02
Vigorous	*r* = − 0.15	*r* = 0.04	*r* = 0.03	*r* = − 0.02	*r* = − 0.15	*r* = − 0.12
Moderate or Vigorous	*r* = − 0.16	*r* = 0.07	*r* = 0.06	*r* = 0.05	*r* = − 0.12	*r* = − 0.08

^*^ = significant at *α* = 0.05; ** = significant at *α* = 0.01; *** = significant at *α* = 0.001

## Discussion

This study adds to our understanding of how physical activity is associated with subsequent mood. Importantly, while previous studies have investigated objectively measured physical activity impacts on mood, this study examines the effects of activity type and activity importance. Accelerometry measures do not provide contextual information about the type of activity, so previous studies have remarked on the difficulty of disentangling the importance of activity type (Poole et al., 2011), which this study helps to address. Additionally, the study was conducted among an ethnically and socioeconomically diverse population, which strengthens its generalizability.

### Within-person

Results from this study suggest that within-person, more sedentary time is associated with subsequent decreased positive affect, while greater physical activity is associated with subsequent increased positive affect. The negative relationship between sedentary activity and positive affect is consistent with previous EMA studies (Smith et al., 2020; Wen et al., 2018)**.** The positive relationship between positive affect and physical activity was found for light, moderate, and vigorous levels of activity. This finding is consistent with previous EMA studies where higher levels of positive affect were found after engagement in moderate-vigorous physical activity (Dunton et al., 2014; Liao et al., 2015; Wen et al., 2018). No significant associations were found between physical activity and negative affect. These null findings are consistent with a meta-analysis of 12 studies that also examined affective responses from physical activity and found no significant relationship between light physical activity and negative affect (Wiese et al., 2018). Numerous meta-analyses across different patient populations demonstrate a relationship between physical activity and the reduction of fatigue (Juvet et al., 2017; Oberoi et al., 2018; Razazian et al., 2020). Activity type and activity importance are shown to have an effect on both positive affect and fatigue, where the types of activities people engage in and the perceived importance of the activities influence the change in positive affect and fatigue that is caused by differing levels of physical activity. In other words, the associations between both physical activity and positive affect and physical activity and fatigue are greater when activity type and activity importance are considered.

The low correlations are consistent with other studies examining short-term, momentary relationships (Bennett et al., 2020; Yang et al., 2021). Additionally, while the effect sizes for these relationships are small, they should be considered in the context of a lifetime. Experiencing slight fluctuations in mood on any given day may not seem significant, but these experiences have the potential to be repeated countless times throughout the lifespan. Thus, though the momentary effects are marginal, the overall effect on mood and well-being may be substantial.

### Between-person

Between-person, people that spent more time in light activity over the study period also typically had higher average stress over the study period. This may be because light activities may include running errands or other hassles and stressful events that occur in people’s everyday lives that may lead to increased stress states. Previous studies have had mixed results regarding light physical activity and stress. Similar to this study, Jones et al. (2017) found that higher light activity was associated with higher stress in real-time. These findings are in contrast to an earlier study that did not find any associations between objectively measured physical activity and stress (Poole et al., 2011). The participant sample from this study is closer in similarity to Jones et al. (2017), which may explain the differing results.

### Limitations

Despite the advantages of using accelerometry to objectively measure activity level and electronic EMA survey methods to assess well-being, this study had a few limitations. To start, the sample consisted of individuals who had type 1 diabetes and had the added experience of participating in the study amidst the COVID-19 pandemic and its related social distancing effects during data collection, hence their experiences may differ from the general population. Furthermore, since the participants were limited to particular geographic areas (Los Angeles and New York), the results may not be applicable to other populations in other areas of the United States or around the world. Replication of this protocol with larger, more diverse samples is needed to confirm the relationships suggested by this study. Additionally, the construct of well-being is a broad, multidimensional concept and all aspects of well-being were not covered in this study. Well-being measures were limited to those included in the overarching study.

## Conclusions

This study provides evidence that positive affect is predicted by previous activity and this relationship is still pertinent even when adjusting for the different activities that people were engaged in. Ultimately, when people were more active, they were in a better mood. Additionally, when people were engaged in activities that they enjoy, this also led to a better mood. There still exists an independent effect that suggests a portion of the improved mood was derived from purely the physical activity component and some portion comes from the activities that are considered important and meaningful.

This study also suggests that while people may experience increased positive affect and reduced fatigue after engaging in physical activity, people who had increased light physical activity also reported higher stress states. Results from this study highlight the value of engaging in meaningful activities to improve mood and reduce fatigue. These findings have implications for the timing of short-term interventions, such as just-in-time adaptive intervention approaches. For example, one direction that the findings from this study may serve to contribute to future interventions is through personalized communication which informs individuals about the types of occupations that may have improved their emotional well-being in the past based on their data. For healthcare practitioners who work directly with patients, it is suggested that physical activity recommendations consider the unique needs and characteristics of the patient, including which activities are important and meaningful for them. The promotion of physical activity should be part of an integrated approach to enhance meaningful occupations and healthy lifestyle behaviors.

## Data Availability

The reported data are available to share upon request.
